# A critical review on sustainable hazardous waste management strategies: a step towards a circular economy

**DOI:** 10.1007/s11356-023-29511-8

**Published:** 2023-09-19

**Authors:** Ashutosh Kumar, Amit K. Thakur, Gajendra Kumar Gaurav, Jiří Jaromír Klemeš, Vishal Kumar Sandhwar, Kamal Kishore Pant, Rahul Kumar

**Affiliations:** 1 Department of Chemical Engineering, Indian Institute of Technology, Delhi, New Delhi 110016 India; 2 School of Chemical & Biotechnology, SASTRA Deemed to Be University, Tirmalaisamudram, Thanjavur, Tamil Nadu 613401 India; 3 Department of Chemical Engineering, Energy Cluster, University of Petroleum and Energy Studies, Dehradun, 248007 Uttarakhand India; 4 Sustainable Process Integration Laboratory, Faculty of Mechanical Engineering, SPIL, NETME Centre, Brno University of Technology, VUT Brno, Technická 2896/2, 616 69 Brno, Czech Republic; 5 Department of Chemical Engineering, Parul Institute of Technology, Parul University, Vadodara, Gujarat 391760 India

**Keywords:** Environmental remediation, Integrated treatment, E-waste, Industrial waste, Recycling, Sustainability, Waste-to-energy, Waste treatment technologies

## Abstract

Globally, industrialisation and urbanisation have led to the generation of hazardous waste (HW). Sustainable hazardous waste management (HWM) is the need of the hour for a safe, clean, and eco-friendly environment and public health. The prominent waste management strategies should be aligned with circular economic models considering the economy, environment, and efficiency. This review critically discusses HW generation and sustainable management with the strategies of prevention, reduction, recycling, waste-to-energy, advanced treatment technology, and proper disposal. In this regard, the major HW policies, legislations, and international conventions related to HWM are summarised. The global generation and composition of hazardous industrial, household, and e-waste are analysed, along with their environmental and health impacts. The paper critically discusses recently adapted management strategies, waste-to-energy conversion techniques, treatment technologies, and their suitability, advantages, and limitations. A roadmap for future research focused on the components of the circular economy model is proposed, and the waste management challenges are discussed. This review stems to give a holistic and broader picture of global waste generation (from many sources), its effects on public health and the environment, and the need for a sustainable HWM approach towards the circular economy. The in-depth analysis presented in this work will help build cost-effective and eco-sustainable HWM projects.

**Supplementary Information:**

The online version contains supplementary material available at 10.1007/s11356-023-29511-8.

## Introduction

Socio-economic development and the pursuit of better livelihoods are essential to the progress of a country. The globalisation of trade, commerce, and tourism is directly linked with the socio-economic status and lifestyle of the people. Industrialisation and urbanisation are the common features of financial development and economic growth. It leads to a dramatic increase in the generation of hazardous waste (HW). HW has been classified as waste with one or more intrinsic chemical and physical characteristics: toxicity, ignitability, reactivity, and corrosivity (EPA 2016). The HW can be dangerous to people or the environment. In contrast, non-hazardous waste does not directly harm people or the environment, like cardboard, glass, plastic, rocks, metals, and food scraps. The United Nations Environmental Programme (UNEP) has indexed waste in the category of hazardous material if they acquire one or more of the characteristics that may lead to the consequences (Saeidi-Mobarakeh et al. 2020), such as:

(i) Fires during routine management
(ii) Corrosive upon exposure to air, or in some particular environment
(iii) Chemical reactions that lead to toxic gas emissions into the atmosphere
(iv) Long-term environmental, geological, and ecological disaster effect

The EPA has established a Toxics Release Inventory (TRI) database in which more than 600 potentially hazardous chemicals are listed (DeVito et al. 2015). On the other hand, hazardous waste is garbage that can be dangerous to people or the environment, like things that can burn easily, poisonous materials, things that react with other substances, or things that can corrode.

**Fig. 1:**
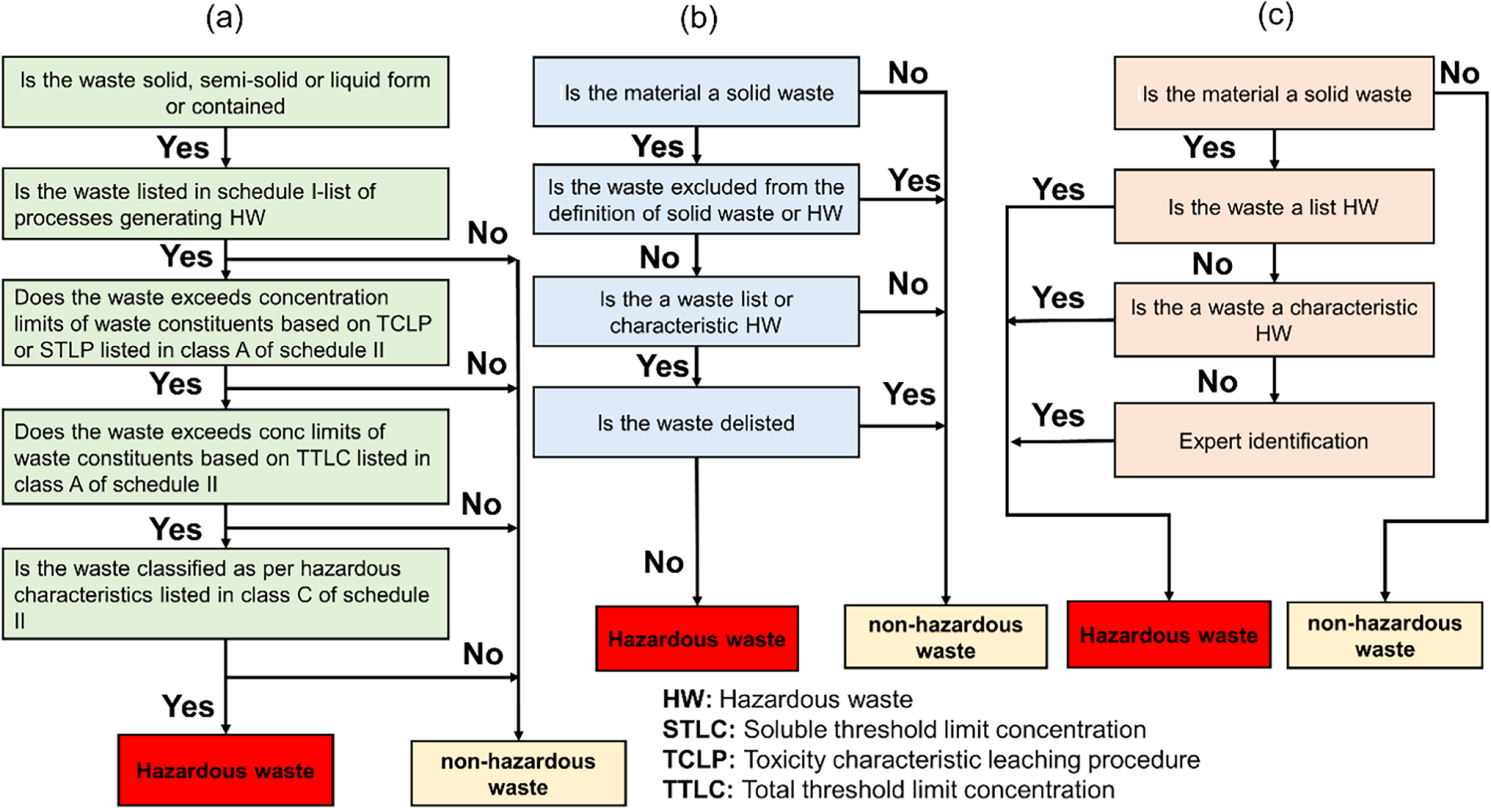
The HW identification process by **a** hazardous and other waste rules, 2016, India, **b** Resource Conservation and Recovery Act Subtitle C of USA, and **c** China GB 5085.7–2007 identification standard (CNMEE 2019)

The identification and classification of HW are essential to ensure their effective management. The identification process also varies from country to country. In the USA, the Resource Conservation and Recovery Act (RCRA) Subtitle C is commonly used to identify HW (USEPA 1976). In India, the hazardous and other waste rules—2016 (HWM rules-2016, 2019) is the identification standard, whereas China uses GB 5085.7 identification standard for HW. The various steps for the preliminary identification of the HW are described in Fig. 1, as per the hazardous and the other waste rule—2016, India (Fig. 1a), RCRA Subtitle C, USA (Fig. 1b), and GB 5085.7 identification standard, China (Fig. 1c). The classification of HW is not unique and varies from country to country. For example, in China, HW is classified as household hazardous waste (HHW), industrial hazardous waste (IHW), and medical waste (MW) (Duan et al. 2008). Most developing countries classify HW as industrial hazardous waste (IHW) and medical waste.

Sustainable HWM is a must for a safe, clean, and eco-friendly environment. This can be achieved by implementing policies and employing environmentally acceptable strategies (Safdar et al. 2020). Some of the common strategies for sustainable waste management (Girondi et al. 2020) include recycling (Alawa et al. 2022), composting (Ayilara et al. 2020), incineration (Li et al. 2019b), gasification (Valdés et al. 2020), and pyrolysis (Li et al. 2021b). This will ensure environmental protection, a green economy, health, safety, and social security. The above waste management strategies can be arranged according to their preferences in the HWM hierarchy. The HW treatment technologies employ physical, chemical, and biological methods. HW’s cost-effective and efficient management may require integrating several technologies. The HWM is a complex and challenging task. It is a big challenge to build cost-effective, sustainable, and environment-friendly industrial projects for the HWM.

**Fig. 2:**
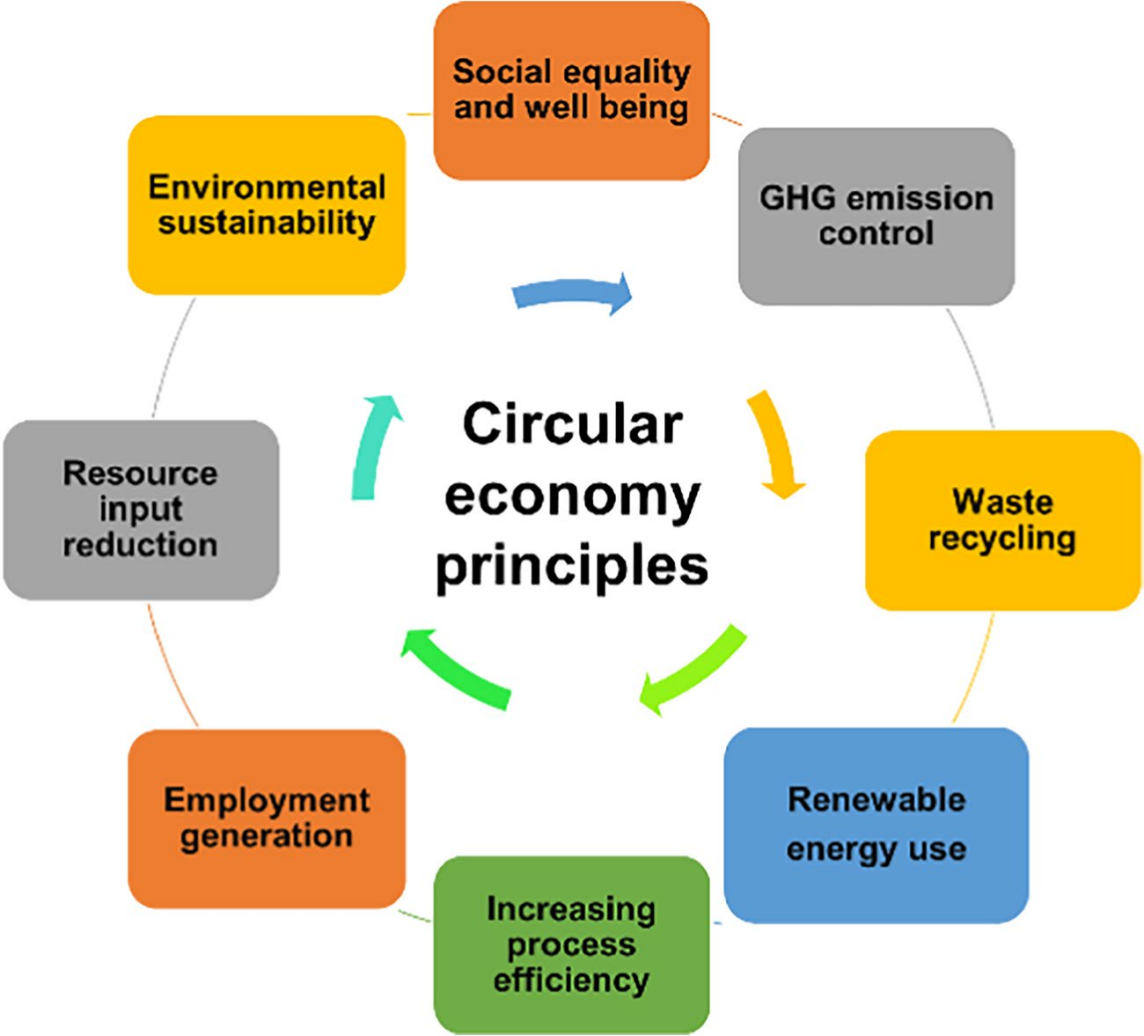
Principles of the circular economy for waste management (Corona et al. 2019)

The circular economy (CE) means producing and utilising things in a way that is good for the environment. It follows the principle that each product is used again and again and nothing is wasted. The CE gives equal importance to economic prosperity, environmental sustainability, and social and individual well-being (Velenturf and Purnell 2021). Different stakeholders and sectors understand the idea of a CE in different ways. The European Union (EU) follows the 10 R principle, redesigning technology, repair, refrain, repurpose, reduce, remanufacture, re-mine, recover, recycle, and reuse (Van Fan et al. 2021). However, most of the definition of CE depicts the idea of 3R principle using the “reduce, reuse, recycle” approach. Figure 2 depicts the main guiding principle of CE for waste management.

Several reviews related to different aspects of HWM have been published in the recent past. Some of the studies related to other elements of HWM published between 2018 and 2022 are summarised in Table 1. Most recently published reviews mainly cover the impact of specific HW on the environment and public health. Many studies are focused on the management of e-waste and medical waste. These reviews mainly focus on managing HW in specific regions/countries. There is a lack of published literature on the broader picture of global hazardous waste generation (from many sources). This review is focused on the generation and composition of waste, including hazardous waste. The major policies, legislation, and international conventions related to HWM are summarised and mentioned in Table S.3. The environmental and health impact of HW is discussed. The paper critically discusses common strategies, waste-to-energy conversion techniques, treatment technologies, suitability, advantages, and limitations. A roadmap for future research work is proposed, and the waste management challenges are discussed.

**Table 1:** A summary of recent reviews related to different aspects of HWM and their salient outcomes

Main review aspects	Region/country	Comments/salient outcomes	Reference
The current status of HWM, policies, distribution, and treatment in China	China	The HW utilisation rate depends on economic development and technological advancement. The HW utilisation was higher in the southeast coastal cities than in the inland cities, and the interprovincial transportation of HW was proposed	(Su et al. 2022)
Medical waste (MW) disposal techniques and management during COVID-19 emergency	China	Based on environmental risk and technical adaptability, six emergency MW disposal technologies are proposed in order of priority (1) portable microwave sterilisation, (2) portable steam sterilisation, (3) movable incineration, (4) co-incineration with hazardous waste, (5) co-incineration with MSW, and (6) co-disposal in cement kilns	(Zhao et al. 2022)
HW impact on environment and health, HWM strategies, recycling	Not focused on any region/country	Different sources of waste products and management strategies were discussed. The gap between waste management strategies and eco-sustainability was highlighted. Lack of awareness of e-waste, especially in developing countries, and future perspectives of waste management were discussed	(Zhang et al. 2022b)
E-waste generation and management	Asia–Pacific region	Continuous technological modernisation, poverty, and lack of integrated waste management framework are the main reasons for the increasing generation of e-waste in developing countries. Implementing extended producer responsibility and 3R (reduce, reuse, and recycle) policies were recommended. The review is limited to Asia–pacific countries	(Andeobu et al. 2021)
Radioactive waste generation and disposal	Not focused on any region/country	A long-term radioactive waste management strategy/storage system must be devised. Such a strategy/system must be permanent and work without any supervision of future generations	(Darda et al. 2021)
E-waste recycling, the health impact of e-waste	Not focused on any region/country	A sustainable e-waste management model was proposed based on sustainable production, recycling, and sustainable use	(Ahirwar and Tripathi 2021)
A bibliographic review on the impact of hazardous medical waste related to COVID-19 on the environment and society	Not focused on any region/country	A collective human effort would be required to create COVID-19 hazardous medical waste management methods. Such methods will ensure transit security, reduce material and labour costs, and sustainably waste utilisation. The review was based on the Web of Science database only	(Bucătaru et al. 2021)
Drivers-Pressures-State-Impact-Response (DPSIR) framework of HWM	China	The future circular economy can be achieved by followings (a) providing support to HWM industries; (b) consideration of the HWM sector as a crucial component of the planning and layout of energy conservation, environmental protection, and sustainable development; and (c) utilising legal mechanisms to compel the disclosure of HW data while also relying on big data and other cutting-edge technology tools	(Kanwal et al. 2021)
Impact of hazardous waste on environment and health	Not focused on any region/country	Sorting, storage, transportation, and processing are essential to waste management strategies	(Exposto and Sujaya 2021)
Identification of HW, impact on public health, recycling, HWM	African countries	A sustainable waste management strategy would benefit significantly from the involvement of all stakeholders, including producers, managers, decision-makers, waste processors, and the formal and informal sectors. Lack of infrastructure, data and awareness, poverty, and poor enforcement of laws are significant challenges to waste management decisions	(Akpan et al. 2020)
E-waste management policy and practices	African countries	The African countries should work with the developed nation and adopt recycling and extended producer responsibility schemes. The public awareness programme is vital	(Bimir 2020)
Recovery of heavy metals from e-waste, laws and regulation, e-waste management strategies	Global	Pyrometallurgy, hydrometallurgy, and electrochemical methods are commonly used to recover valuable metals from e-waste. Leaching gold using a pinch of acid, vinegar, and an oxidant is an economically and environmentally sustainable method. Biotechnology is a promising approach to metal recovery. Bioleaching efficiency improvement and microbial consortia development are the major challenges with the biological processes	(Islam et al. 2020)
E-waste management, the impact of e-waste on the environment, and public health	African countries	E-waste management in developing nations is often unsustainable. Awareness/capacity-building and policies will reduce the import of e-waste in Africa	(Asante et al. 2019)
HHW management, e-waste management	Asian countries	Except for e-waste, HHW generation of Asian countries is not influenced by the country’s financial status. Developed countries generally ship their e-waste to less developed countries. The lower-middle-income group countries have the lowest awareness about HHW risks	(Manggali and Susanna 2019)
Hazardous solid waste management, legislation, best practices	India	The “polluter pays concept” must be emphasised in HWM policy. The polluter should pay the penalty as well as the costs of pollution control. Effective waste management may also increase resource security and improve environmental and public health	(Karthikeyan et al. 2018)
E-waste management, legislation, technology	India	Better implementation of the e-waste regulations is required to manage e-waste in India properly; environmentally sound technology with a high yearly recycling capacity is needed	(Awasthi et al. 2018)

## Waste generation and composition

**Fig. 3:**
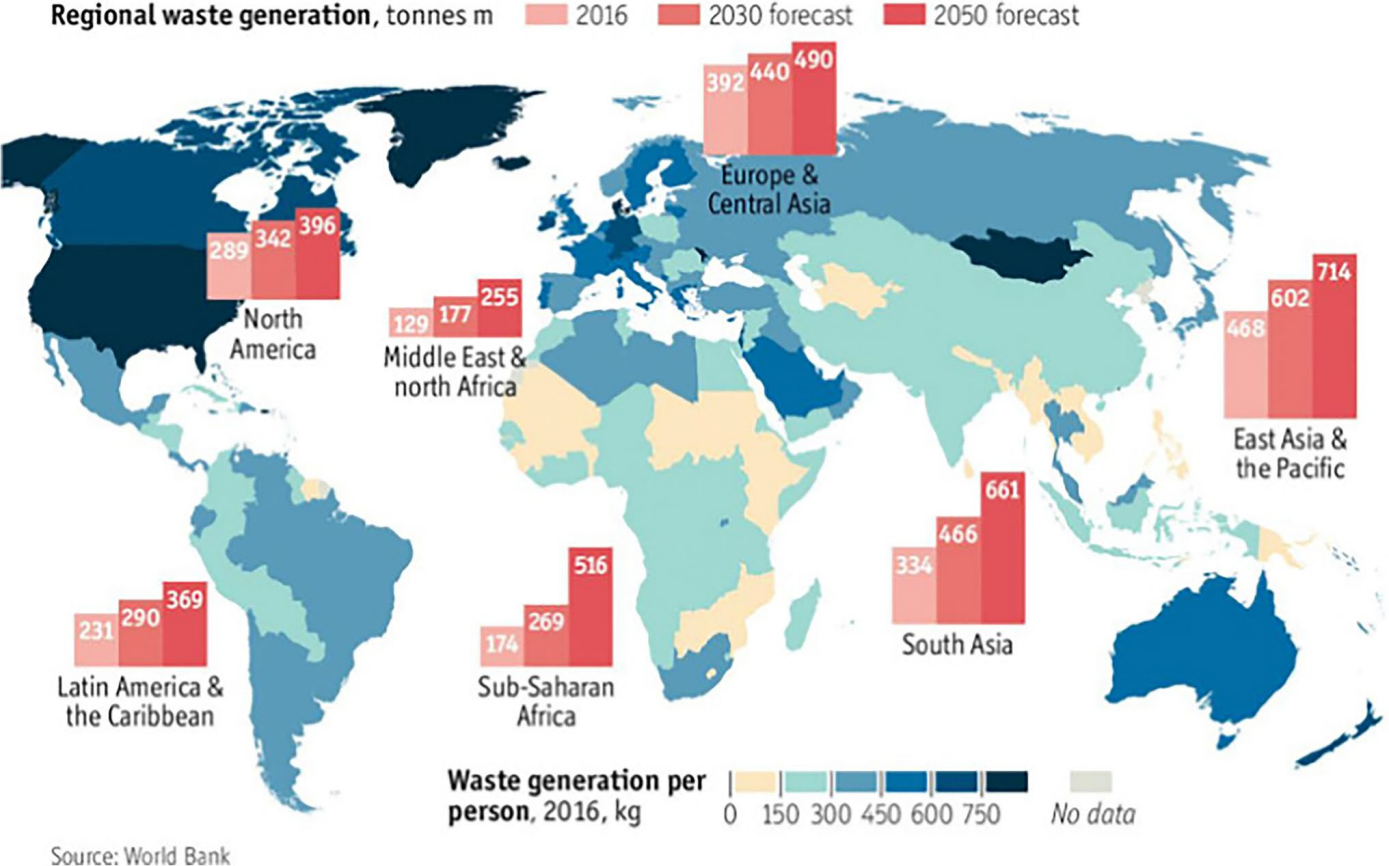
The global trend of waste generation and forecast for 2030 and 2050 (Economist 2018) (Kaza et al. 2018)

The amount of waste generated from different sources varies from country to country. This is directly linked with industrialisation, urbanisation, and economic development. The countries with high incomes and economies are urbanised and subsequently produce more waste per capita compared to the low-income countries (Kaza et al. 2018). The global and regional waste generation patterns and projections from 2016 to 2050 are shown in Fig. 3. Globally, 2.01 billion Mt/year of municipal solid waste (MSW) is generated and anticipated to increase by 2.59 billion Mt/year and 3.40 billion Mt/year by 2030 and 2050, respectively (Kaza et al. 2018). The amount of MSW produced per day can vary between 0.11 and 4.54 kg, and this variation is dependent on geography and the level of development (Roy et al 2022a). Asia generates one-third of the global waste, with China (0–0.49) kg/capita/day and India (0.50–0.9) kg/capita/day making substantial contributions.

**Fig. 4:**
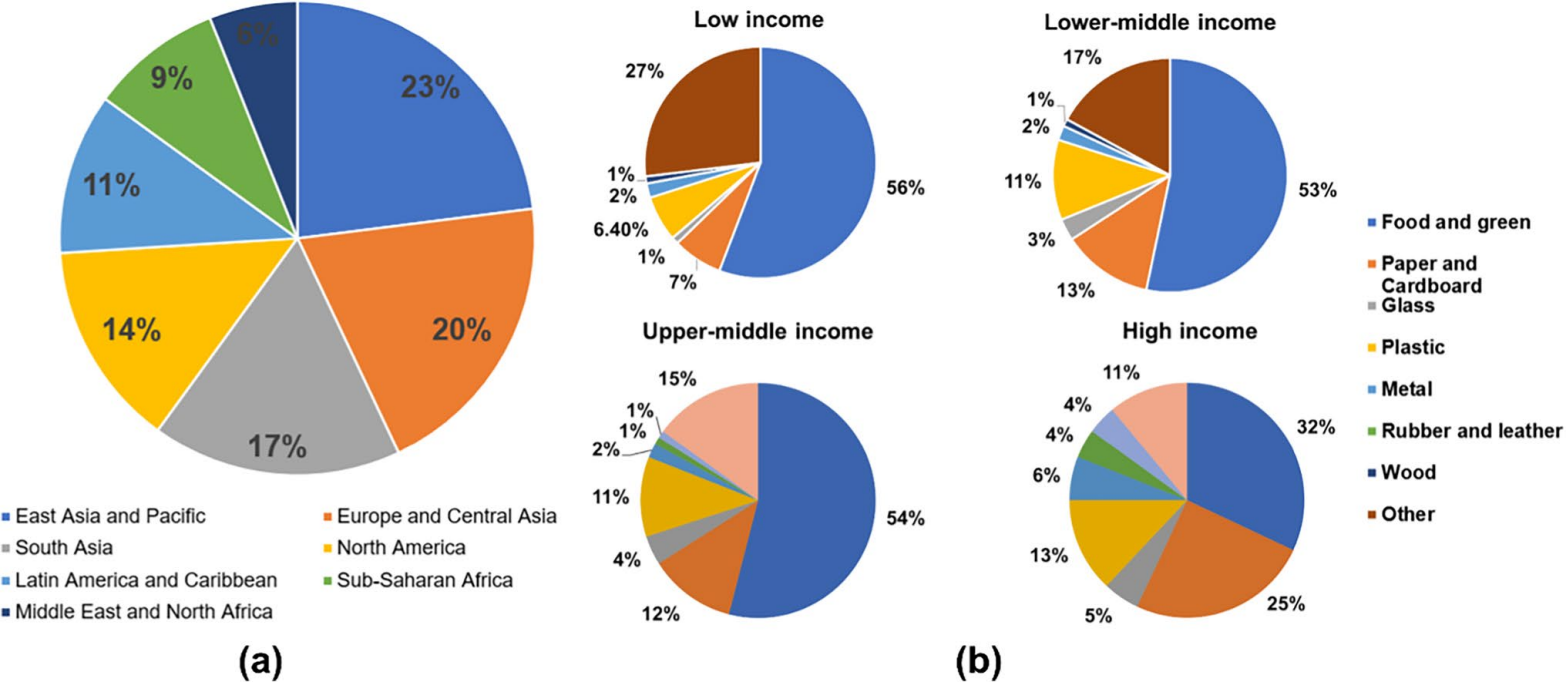
Global waste generation by **a** region and **b** income level (Kaza et al. 2018)

**Fig. 5:**
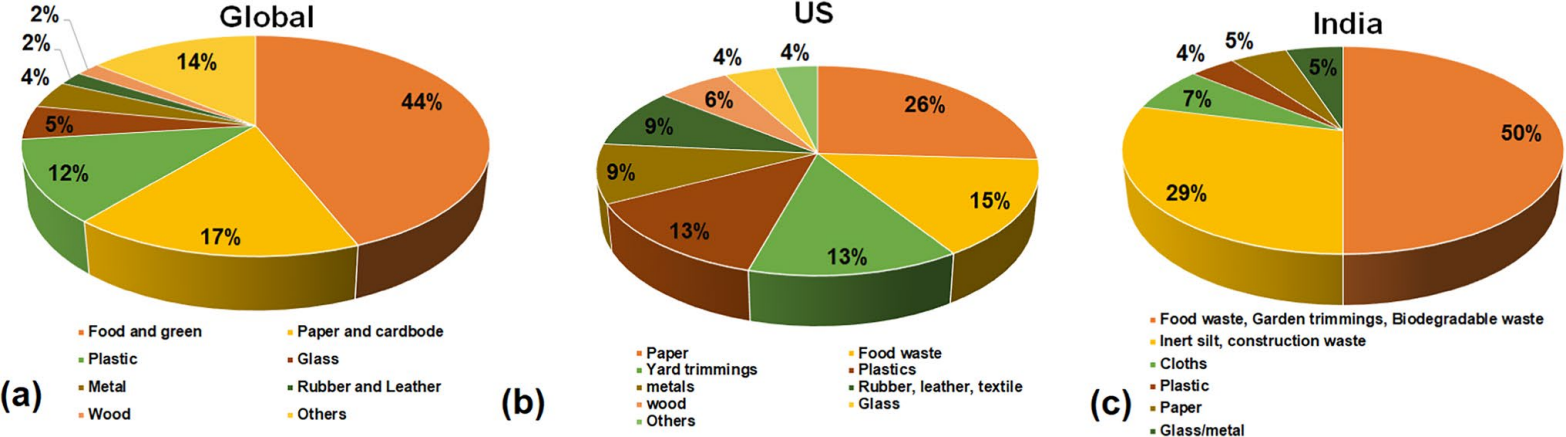
The composition of municipal solid waste **a** global (Kaza et al. 2018), **b** USA (EPA 2016), and **c** India (CPCB | inventory 2022)

A typical MSW contains hazardous waste such as metals (1.1–2.2%), polyethylene (2.8–4.3%), glass (0.5–1.1%), and rubber (0.1–1.4%). The other non-hazardous fractions in MSW are food waste (68.3–81.1%), paper (7.2–10.7%), textile (1.3–2.2%), and others (4.5–10.4%) (Roy et al. 2022a). The global waste generation by region is illustrated in Fig. 4a. The countries in East Asia and Pacific, South Asia, Central Asia, and Europe produce 60% of the global waste. The Sub-Saharan African regions generate the least amount of waste (only 9% of the global). The global waste composition by income level is shown in Fig. 4b. It is observed that the percentage of paper, plastic, and electronic waste increases with the improvement in income level. The percentage of food and green waste decreases with the progress of income level. Dry waste (plastic, paper, metals, and glasses) makes up a major portion of MSW in high-income countries. However, in low-income countries like Bangladesh, India, and Pakistan, the proportion of food- and agriculture-based refuse in MSW is close to 57–60%, increasing the organic content of MSW (Roy et al. 2020a). Only 20% of the MSW in low-income nations is made up of recyclable materials (Roy et al. 2022c). The easily recyclable dry waste (plastic, paper, metals, and glasses) makes up a sizable portion of MSW in high-income nations. A comparison of the global municipal solid waste composition with the MSW composition of the USA and India is presented in Fig. 5. It is observed that food waste, paper, and cardboard are the key components of the MSW. A recent report estimates that more than 150,000 Mt solid waste was generated in India, out of which about 90% was collected as a waste product (Shrivastava 2019). The survey also reports that only 20% of the total accumulated waste was processed, and the remaining 80% was dumped in landfills. Several factors, such as lack of advanced technology, insufficient treatment facilities, and limitations in regulation, lead to the ineffective management of HW. The inefficient transport services, spatially limited collection regions, open burning, illegal dumping, and lack of proper recycling (Kula et al. 2023) and disposal facilities are major factors leading to the mismanagement of the HW. The mismanagement of HW leads to land, water, and air pollution (Ferronato and Torretta 2019). Industries are the backbone of economic development, and continuous generation of HW is unavoidable (Jiang et al. 2021). It can be reduced by implementing integrated policies, regulations, and technological advancements. For the sustainable socio-economic development of nations, a holistic approach should be identified and implemented to practise hazardous waste management (HWM) (Bing et al. 2016).

In the current global pandemic situation due to COVID-19, it has even become more important to manage municipal solid waste and medical waste to control the spread of the virus (Klemeš et al. 2020c). The amount of plastic waste significantly increased during COVID-19. The quantity of plastic waste generated globally rose by approximately eight million tons between 2018 and August 2021 due to the increase in the number of COVID-19 (Choudhary et al. 2023) cases. Asia was solely responsible for 46% of this increase (Peng et al. 2021). China reported a significant increase in medical waste post-COVID-19 outbreak (6606.8.8 tonnes/day) compared to pre-COVID-19 (4902.8 tonnes/day) (Ma et al. 2020), whereas in the USA it increases from 2.5 to 5.0 Mt/month (Ilyas et al. 2021).

### Hazardous waste

Industrialisation plays a significant role in improving a nation’s economy and, subsequently, the lifestyle of its fellow citizens. It has caused severe consequences due to excessive HW generation and ineffective management. A considerable amount of industrial waste generated during the manufacturing process is hazardous. The industrial waste can be classified as solid, liquid, and gaseous waste. Central Pollution Control Board (CPCB), India, mentioned 64 polluting industries as “Red Category” based on emissions of hazardous waste, including small-, medium-, and large-scale enterprises.

The HW is generated from discarded consumer products, chemicals, pharmaceuticals, pesticides, personal care products, electronic products, industrial by-products, sludge, and waste oils. The United Nations (UN) estimates that around 68% of the world’s population will get urbanised by the year 2050 (Knickmeyer 2020). As per recent research by the United Nations Environment Programme (UNEP), the global production of HW amounts to roughly 400 million tons annually, which translates to roughly 60 kg per person around the world and this is continuously increasing. Within the span of a single generation, the production of synthetic chemicals has increased by 40,000%, from 1 to 400 million tons (The World Counts 2023). Some developed countries produce substantial amounts of hazardous waste, while others produce very small amounts (Gautam et al. 2019). The USA, China, and some states of the European Union (EU) are the major producers of HW. Hazardous waste classification is complex, and there is no universally accepted classification. Even within the same country, different regulatory programmes classify HW differently. The classification and definitions of hazardous waste/products may vary over time due to climate change (Rabbani et al. 2019). The HW consists of solid and liquid waste from chemical industries, hospitals, households, mining, construction, etc. The emissions from factories and industries contribute to gaseous HW. Industrial effluents discharge into rivers, and aqueous bodies heavily contribute to surface water pollution. The following sections briefly describe three major types of HW: household hazardous waste, industrial hazardous waste, and e-waste.

Household hazardous waste (HHW) is another major issue that needs to be addressed properly. The US EPA defines HHW as “leftover household products that can catch fire, react, or explode under certain circumstances, or that are corrosive or toxic” (Household Hazardous Waste US EPA). In Europe, the HHW is defined as “such waste that could potentially increase the hazardous properties of municipal solid waste when landfilled, incinerated, or composted”. The HHW mainly includes paints, batteries, detergents, personal care products, pharmaceuticals, insecticides, and house cleaning chemicals (Inglezakis and Moustakas 2015). The percentage of HHW in MSW varies between 1 and 4% (Vallero et al. 2019). The rate may slightly differ from one country to country. A recent study (Manggali and Susanna 2019) estimates that the HHW generation is 0.038 kg/person/day in upper-middle-income countries and 0.028 kg/person/day in lower-middle-income countries of the Asian region. All HHW does not have an immediate harmful effect on human beings and the environment. Numerous chemical products like house cleaners may be hazardous to specific species or environments. The products are much vulnerable and poisonous to children and pets (Franklin and Rodgers 2008). A list of personal care and cleansing products frequently used in daily life or household purposes such as toilet cleaners, glass/window cleaners, beauty care products, medicines, and insects killing products (Koushki and Al-Humoud 2002). Most common people are unaware of the hazardous nature of the products they use. The proper labelling of the packaging materials is essential to identify hazardous products. The awareness of the hazardous nature of HHW is vital to avoid their potential health and environmental consequences.

Industries use different hazardous chemicals to meet their productivity and yield, and as a result, a massive amount of HW is generated. Globally, the manufacturing industry is the primary IHW source. IHW mainly originated from thermal power plants, integrated steel and iron mills, mining operations, cement industries, paper industries, fertilisers, textiles, and other allied industries. In China, the chemical industry is the largest producer of IHW, followed by the non-ferrous metal mining and smelting industries. These two account for 45% of the IHW in China (Su et al. 2022). Table S.1 (supplementary material) summarises the HW generated from different industries.

**Fig. 6:**
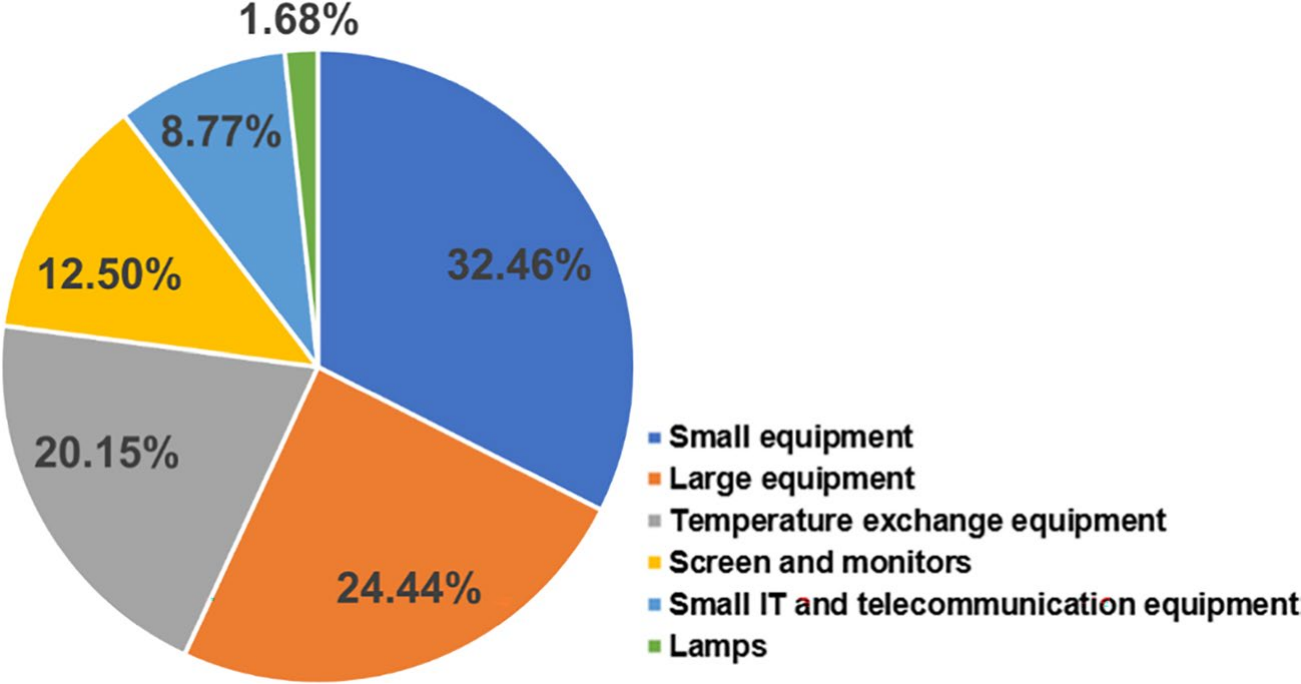
A typical composition of e-waste generated in 2019 (Forti et al. 2020)

Electrical and electronic equipment (EEE) and technology have become integral to life. The growth and demand of the EEE have led to a significant increase in the volume of e-waste. As per a recent report (Forti et al. 2020), 53.6 Mt of e-waste was generated globally in 2019, with 21% increments in only 5 years. The global e-waste discarded product with a battery or plug is expected to reach 74 Mt by 2030 and 120 Mt by 2050 (Yu et al. 2023). The e-waste statistics for the year 2019 are summarised in Table 2. In 2019, Asia generated the highest amount of e-waste, and Europe generated the highest e-waste per capita. In Asia, China is the highest generator of e-waste (10.1 Mt), followed by India (3.2 Mt), Bangladesh (3.1 Mt, own generation—0.6 Mt, imported—2.5 Mt), and Japan (2.5 Mt) in 2019 (Fu et al. 2018; Kumar et al. 2022; Roy et al. 2022c). Generally, 60.2% of e-waste consists of various precious metals (such as gold, silver, aluminium, iron, copper, and platinum), with plastic materials (15.2%), plastic-metal mixture (5%), screens and tubes (12%), printed circuit boards (PCBs, 2%), cables (2%), and other components (Roy et al. 2022b). Figure 6 shows the composition of e-waste generated in 2019. The e-waste contains several hazardous materials such as toxic metals (Hg, Pb, Cg, Cr, etc.), chemicals (halogenated flame retardants, polybrominated biphenyls, polybrominated diphenyl ethers, etc.), and non-toxic (plastics, cables, etc.) that can cause several environmental and health concern upon their mobilisation (Sengupta et al. 2022). A substantial amount of the precious metals can be recovered and re-enter the manufacturing process of EEE and can establish a sustainable circular economy.

**Table 2:** Global statistics of e-waste for 2019 (Forti et al. 2020)

Regions	E-waste generated (Mt)	E-waste generated per capita (kg)	E-waste documented to be collected and recycled (Mt)
Asia	24.9	5.6	2.9
Europe	12	16.2	5.1
Africa	2.9	2.5	0.03
Americas	13.1	13.3	1.2
Oceania	0.7	16.1	0.06

## Important legislation and conventions

The implementation of legislation is crucial for managing waste generated by industries and households. The US Environmental Protection Agency (EPA), formed in 1970, is involved in the HWM, which emphasises four fundamental requirements: (a) identification of hazardous waste; (b) formulate standards for HW generators, treatment, transportation, collection, storage, and disposal; (c) need of authorisation certificate for HW processing; and (d) establishment of HW tracking system from generation to disposal (Peretz et al. 1997). In 1976, the Resource Conservation and Recovery Act empowered the Environmental Protection Agency (EPA) to regulate hazardous waste. The amendment of previously existing solid-waste management legislation of 1965 was passed through the RCRA (Daniels et al. 1982). The RCRA, 1976, enforced the chemical industry to follow a strict management system for hazardous waste with less emphasis on solid waste and environmental concerns (Daniels et al. 1982). Later in 1984, the RCRA was amended by the Hazardous and Solid Waste Amendment (HSWA), which includes a few silent features such as solid-waste management, regulation of hazardous waste, the prohibition of open dumping, encouraged the implementation of new and advanced technology for resource recovery, reuse, and disposal (Peretz et al. 1997). The most significant limitation of this amendment was the inclusion of small-scale industries such as auto repair shops, medical offices, household waste, and e-waste, which are leading contributors to hazardous waste generators (McGlinn 2000). The principal legislation relating to HWM and their salient features/targets is summarised in Table S.2.

The increasing environmental problem due to HW brought the G8 countries together to jointly agree on the policies of reduction, recycling, and reuse (3R). Subsequently, Japan implemented this policy in the year 2005. The regional 3R forum was launched in Asia in November 2009 in collaboration with the Ministry of the Environment (Government of Japan) and the United Nations Centre for Regional Development (UNCRD) (Ilankoon et al. 2018). The Government of India passed hazardous waste handling and management rules in 1989, which were revised in 2008 under the Environment Protection Act of 1986 (MoEFCC 2015). Under the revised regulations, hazardous waste is classified according to its physio-chemical properties, toxicity, reactivity, corrosive, explosive, and flammable characteristics, which may severely affect the environment and the health of living organisms (MoEFCC 2015). The HW transboundary movement, handling, and management rule of 2008 advocates to have prerequisite authorisation certified by the concerned Pollution Control Committee (PCC)/State Pollution Control Board (SPCB) for collection, treatment, processing, conversion, generation, storage, use, package, transportation, and offering for sale. As India modified and revised the new HWM rules, passed in 2016 in accordance with international standards, covering 3R policies and their disposal in an environment-friendly approach (MoEFCC 2016), the new HW rules also differentiate hazardous and other waste (includes e-waste, scrap metals, plastics, waste tyres, and paper) for the first time.

The countries have different policies and regulations for the HWM. In some countries, there are no specific regulations for the management of HW. In China, most cities have set up a standard policy for collecting HHW. The HHW is processed by a recycling company authorised by the Ministry of Environmental Protection (Tai et al. 2011). In Germany, the HHW are collected using two collection schemes: recycling centres and mobile recycling centres (vehicles that stop at specific locations regularly) (Inglezakis and Moustakas 2015). A 1-day community collection programme in the USA is the most common practice. The municipality collects the HHW on a scheduled day. The USA, Canada, and a few other countries also adopted product stewardship (PS) and extended producer responsibility (EPR) policies for waste management. According to EPR, end-of-life management is the primary responsibility of the manufacturer. The PS policies encourage stakeholders (designers, manufacturers, and consumers) to share responsibility throughout the lifespan of a product. In several Asian countries such as Malaysia, Indonesia, Vietnam, and Cambodia, there is no specific regulation on HHW (Chaib et al. 2014) and consequently, mixing the HW with general household waste and their open burning is a common practice (Manggali and Susanna 2019).

The circular economy (CE) polices have been highly endorsed and discussed, aiming for minimal waste generation and continuous reuse of resources (Klemeš et al. 2020a). Sustainable consumption and production are big concerns globally. There are only a few international agreements and conventions that talk about the CE and other environmental concerns of the CE. For example, the Stockholm Convention on Persistent Organic Pollutants 2001, the International Tropical Timber Agreement 2006, and the Paris Agreement on Climate Change 2015 have covered the elements of CE (Mikichurova and Vlialko 2021). However, at the national level, various countries have developed some policies related to CE. In 2016, Finland made a plan called “A Roadmap to a Circular Economy 2016–2025” in fact Finland made the first plan in the world to switch to a circular economy. Scotland was the first country to join a group called Circular Economy 100, which works together to make new ideas and help the circular economy grow. The Netherlands adopted a plan called “Government-wide Programme for a Circular Dutch Economy by 2050” in 2017. The European Union (EU) has introduced a circular economy (CE) model comprising the 10 R’s hierarchy. The CE 10 R’s prioritise redesigning technology, repair, refrain, repurpose, reduce, remanufacture, re-mine, recover, recycle, and reuse (Van Fan et al. 2021). This waste management policy of the EU provides a regenerative model for the European Green Deal action plan (Van Fan et al. 2021). The CE model’s purpose was to reduce overall waste, recycle more than 50% of the waste by the year 2020, and reuse it in whichever manner possible (Knickmeyer 2020). In the case of Asian countries, China and Japan were the first to introduce the circular economy model nationally (Reike et al. 2018).

Several international agreements are there to protect the environment and public health from HW. The Basel, Rotterdam, Stockholm, and Minamata conventions are multinational environmental agreements summarised in Table S.2. The Basel Convention, which went into effect in 1992, is an agreement among 53 countries to manage the cross-border transportation HW. This focuses on limiting the transfer of HW from developed nations to developing or less-developed nations. However, this convention did not meet its goal of restricting the trade of HW. The convention allows member countries to trade waste with each other. Additionally, the convention lacks a mechanism to guarantee that the importing country has proper waste treatment procedures in place.

## Environmental and health risks

A considerable amount of waste is potentially hazardous to the ecological systems (soil, air, and water) and human health (Xu et al. 2018). The improper handling, storage, treatment, and disposal of HW adversely impact the environment and health. Intake of foul-smelling gas generated from waste (especially hazardous chemicals) can induce serious health problems (Torkashvand et al. 2020) and sometimes even cause death if the intake is significant. In some cases, even minimal doses of hazardous chemicals cause severe health issues.

The illegal disposal of household waste is typical in some developing countries. This leads to large-scale pollution of air, land, and water bodies. In the scenario of the COVID-19 pandemic, the proper treatment and management of waste, especially medical and municipal solid waste, has become even more important to eradicate the spread of the virus. During the pandemic, the Hubei province of China recorded more than a 370% rise in medical waste (Klemeš et al. 2020d). Due to the complete lockdown situation, MSW generation was reduced to 30% (Klemeš et al. 2020d). Plastic waste has increased significantly in volume as most medical kits like PPE, gloves syringe packaging, and others are made up of plastics and have to be single-use to manage the spread of the virus (Klemeš et al. 2020b). Plastics have also entered our food chain. Sustainable plastic waste management is economically and environmentally essential (Klemeš et al. 2020a).

**Fig. 7:**
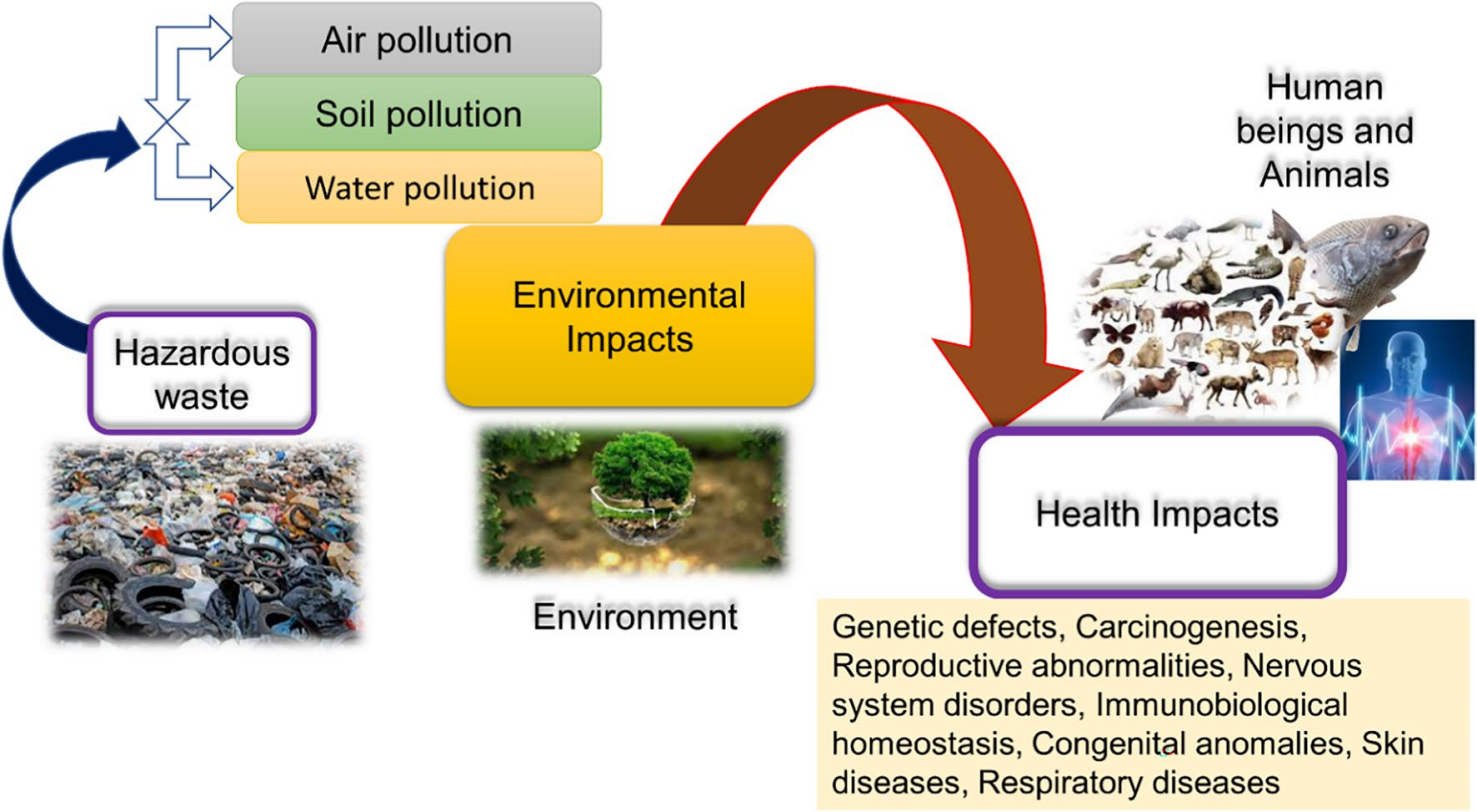
The environmental impact and health impact of the hazardous waste

The uncontrolled exposure of HW to the soil, water, and air leads to various environmental and health issues (Bennett 1988). Several ways, such as skin contact, inhalation, and ingestion, through which one can be exposed to HW. Long-term exposure to such HW may lead to several health risks. The impact of HW on the environment and health is represented in Fig. 7.

## Hazardous waste management

The proper management of HW is inevitable to avoid its potential health and environmental impact. There are several options/strategies for the management of HW. These strategies can be arranged in a hierarchy according to their preference. The typical HWM hierarchy shown in Fig. S.1 demonstrates that the HW reduction at source/prevention is the most preferred strategy, followed by waste minimisation through reuse, recycling, and composting (Wang et al. 2018), recovery/waste to energy, treatment, and disposal. This is a conceptual framework for guiding and ranking waste management decisions. The first stage of the HWM hierarchy encourages stakeholders (industries and communities) to reduce the use of raw materials by maximising efficiency or preventing unnecessary use in manufacturing processes. Reuse and recycling are the second-best strategies of the HW hierarchy. This step allows the stakeholders to avoid spending on the new material and saves energy and resources required to produce the new product. When recycling and reuse are not feasible, waste-to-energy conversion or material recovery is the next preference in the hierarchy. Pyrolysis (Chew et al. 2021), gasification (Hameed et al. 2021), and incineration (Wajda et al. 2022) are the commonly used strategy for waste-to-energy conversion. The treatment of HW using physical, chemical, and biological methods is a preferred strategy when waste-to-energy conversion or material recovery is not a feasible option. Disposal is the least preferred option and is practised only if all other options are not viable. Landfilling is the most commonly used disposal method (Kamaruddin et al. 2017). This is an unsustainable approach because the toxic leachate and emissions from the landfilled waste may continue to impact the environment and public health for a long time. Based on the toxicity level of the HW and its impact on the environment and public health, a combination of waste management strategies may be followed for the sustainable management of HW.

**Fig. 8:**
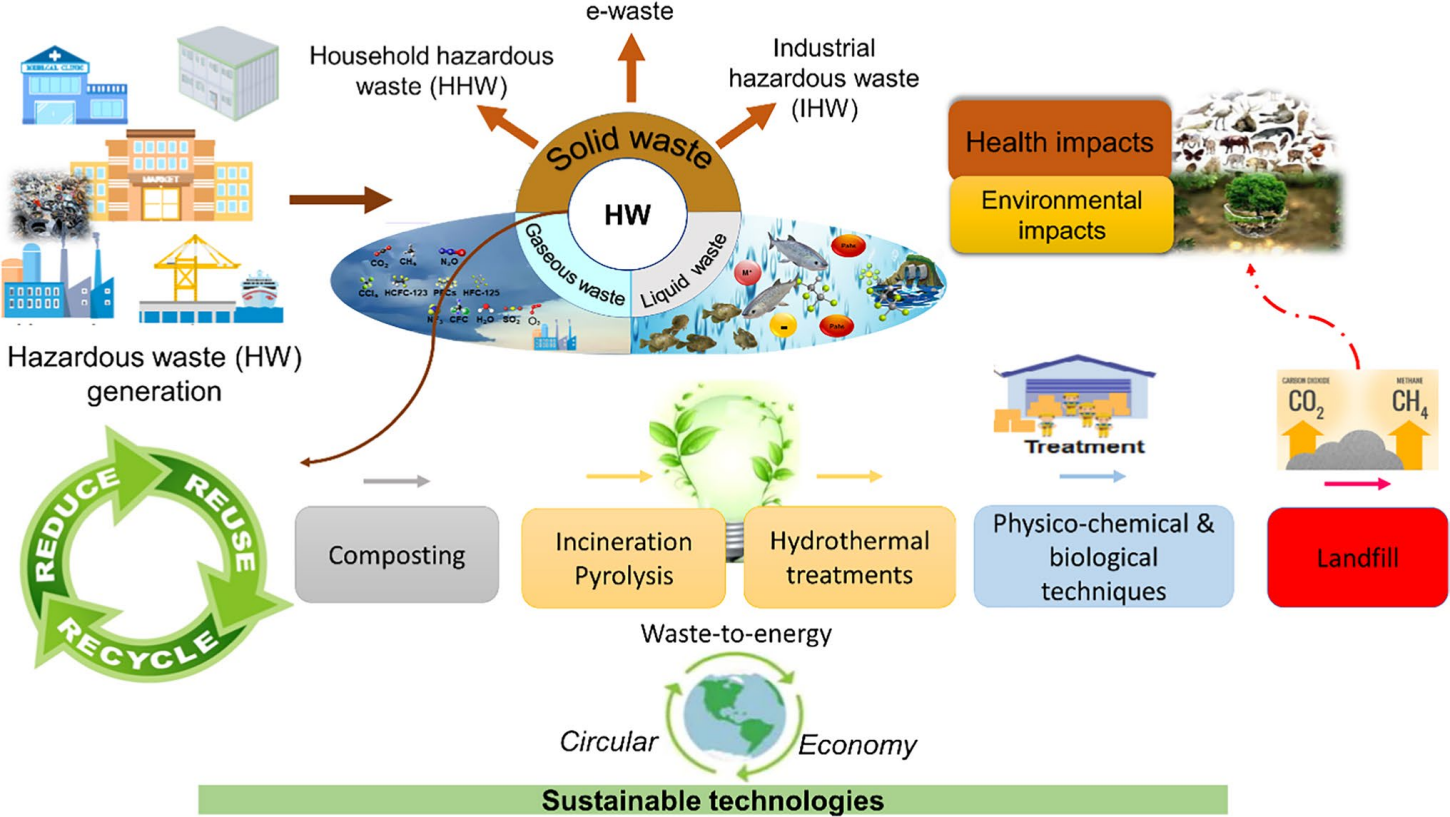
A graphical framework of HW generation, classification, management strategies, and impacts

A graphical framework of HW generation, classification, HWM strategies, and health and environmental impact is described in Fig. 8. In the present scenario, landfilling (Nanda and Berruti 2021) is the most commonly used disposal mechanism. Landfill sites generate two forms of pollutants: leachate and volatile organic compounds (VOCs) as gaseous emissions (Christensen et al. 2001). Dumpsite leachates may penetrate deep into the soil and pollute groundwater. Animals and scavengers can dislocate HW from dumpsites and spread diseases (Jeswani and Azapagic 2020). The incineration of HW waste, such as medical waste, plastics, and rubber, leads to releasing toxic gases into the environment. Leachate emissions from incinerators (Kjeldsen et al. 2002) need much attention for research because, locally, these can severely affect the air quality and contaminate groundwater (Christensen et al. 2001).

### Hazardous waste management strategies

#### Recycling

Recycling is a sustainable HWM strategy to minimise hazardous waste’s harmful impact and make it safe to reuse and later dispose of the waste. Some HW materials, such as plastics, glass components, and e-waste, are of great economic value and can be re-used or re-processed to recover valuable goods. This would help save resources and energy (Kong et al. 2020). Common ways to recycle industrial waste include (a) the breakdown of waste into a valuable product (Savoldelli et al. 2017), (b) resource recovery (Sadala et al. 2019), and (c) reclamation of the valuable products from waste (Ilyas et al. 2021).

Researchers have explored recycling waste materials such as plastics (Dalhat and Al-Abdul Wahhab 2015), ground tyre rubber (Pouranian et al. 2020), glass (Mustakiza Zakaria et al. 2017), cigarette butts (Rahman et al. 2020), and fly ash (Mohammadinia et al. 2017) as aggregate, additives, and filler in asphalt concrete and bitumen for the construction of roads and highways. Sustainable management of hazardous waste cooking oil (WCO) is a challenging task (Gupta and Rathod 2018). The recycling and conversion of WCO into valuable products is a sustainable approach to establishing the WCO-based circular economy (Usmani et al. 2021). The untreated WCO and chemically and biologically treated WCO have been recycled as raw material for the production of value-added products (Goh et al. 2020). In the recent past, many researchers have explored the conversion of WCO into biodiesel using different sustainable technologies (Sahar et al. 2018), such as using eggshell-derived MM-CaO catalyst (Nadeem et al. 2021) and using heterogenous catalyst derived from cork biochar (Bhatia et al. 2020). Recycling is a clean approach to managing HW, but this is a relatively undeveloped technique and often requires high investment costs. An economic analysis should be considered before the implementation of this strategy.

#### Composting

Composting is a promising strategy for managing waste products from different sources (Wei et al. 2020). This strategy treats yard waste, sewage sludge, and agricultural waste with negligible concentrations of hazardous organic substances (Wang et al. 2018). Composting reduces waste size and volume, allowing it to be disposed of more easily. This method is used to handle not only domestic waste but also waste from industrial activities. This aerobic method converts harmful waste into useful bio-fertilisers (Smith and Aber 2018). The bio-fertilisers are subsequently used to improve soil productivity and suppress plant diseases (Sun et al. 2020). Composting also releases toxic gases such as SOx, NOx, and CO_2_ emissions. The emissions from composting are characterised by their high flow rates, low pollutant concentrations, and VOCs. Biofiltration is commonly used to minimise emissions from composting (Hong and Park 2004). The three widely practised composting systems include windrow, aerated static pile (ASP), and in-vessel. Windrow composting (Kong et al. 2018) is an outdoor composting method and uses mechanical aeration (Al-Rumaihi et al. 2020). This method is suitable for waste with less odour emission and requires a large land area and more time for mature composting. Composting with an aerated static pile (ASP) (Michel et al. 2022) involves forcing ambient air through the compost pile. This method is commonly used for MSW and generally requires less land area and relatively less time for mature composting. In the in-vessel composting method (Walker et al. 2009), the composting process is contained within various containers or vessels. This method is suitable for all types of waste and requires a low land area and a short composting period. However, the capacity and performance of the composting process is limited by microbiological components. In a recent review, a guiding perspective for composting models was proposed, which involved the fates of C, N, P, and K and presented a discussion to characterise the fates of C, N, P, and K through modelling (Yang et al. 2021).

#### Incineration

Incineration is a method to burn toxic organic constituents of hazardous waste and reduce waste volume. Incinerators are commonly used to burn hazardous waste for waste destruction/treatment purposes and to recover the material, chemicals, and energy from the waste (Block et al. 2014). There are various types of hazardous waste incinerators (Trinh et al. 2019), namely, rotary kilns (Jiang and Li 2019), fluidised bed units (Azam et al. 2019), and liquid injection units (Anufriev 2021). There are several advantages of incineration over other treatment strategies. The significant benefits of incineration include avoiding groundwater pollution, energy recovery, and using relatively small space to manage waste. This method’s main disadvantages are the emissions of toxic pollutants and higher plant costs. The emission of organic contaminants from solid waste incineration in China was evaluated and established a relationship between the energy benefits and the pollutant emissions (Li et al. 2021a). The authors proposed the energy benefit-to-emission index for organic pollutants to evaluate solid waste management on a local or regional scale. A value of the energy benefit-to-emission index higher than 60 can be used as a benchmark for “profitable” solid waste management (Li et al. 2021a).

#### Landfilling

The landfill is an unsustainable method for the management of non-liquid HW materials. Landfilling is the most commonly used waste management strategy in developed countries. The hazardous waste is separated before landfills to reduce environmental harm. Dumped waste undergoes physico-chemical and biological transformation at the landfill sites. Essential elements for landfilling are landfill liners, soil cover thickness, leachate collection, landfill gas recovery, etc. The weather condition, temperature, moisture, pH, and biodegradable matter are important parameters affecting the emission of landfill gases and the amount of leachate (Nanda and Berruti 2021). Landfilling is a low-cost, straightforward, less labour-intensive, and large-capacity approach. This strategy is most suitable for waste with lower organic and moisture content. This method generates a huge amount of toxic leachate and greenhouse gases. The energy and environmental analysis of landfilling and composting-landfilling of MSW was conducted in Iran (Behrooznia et al. 2018). The investigation revealed that the energy use of composting-landfilling was more than that of landfilling. The composting-landfilling was more eco-friendly than landfilling (Behrooznia et al. 2018).

**Fig. 9:**
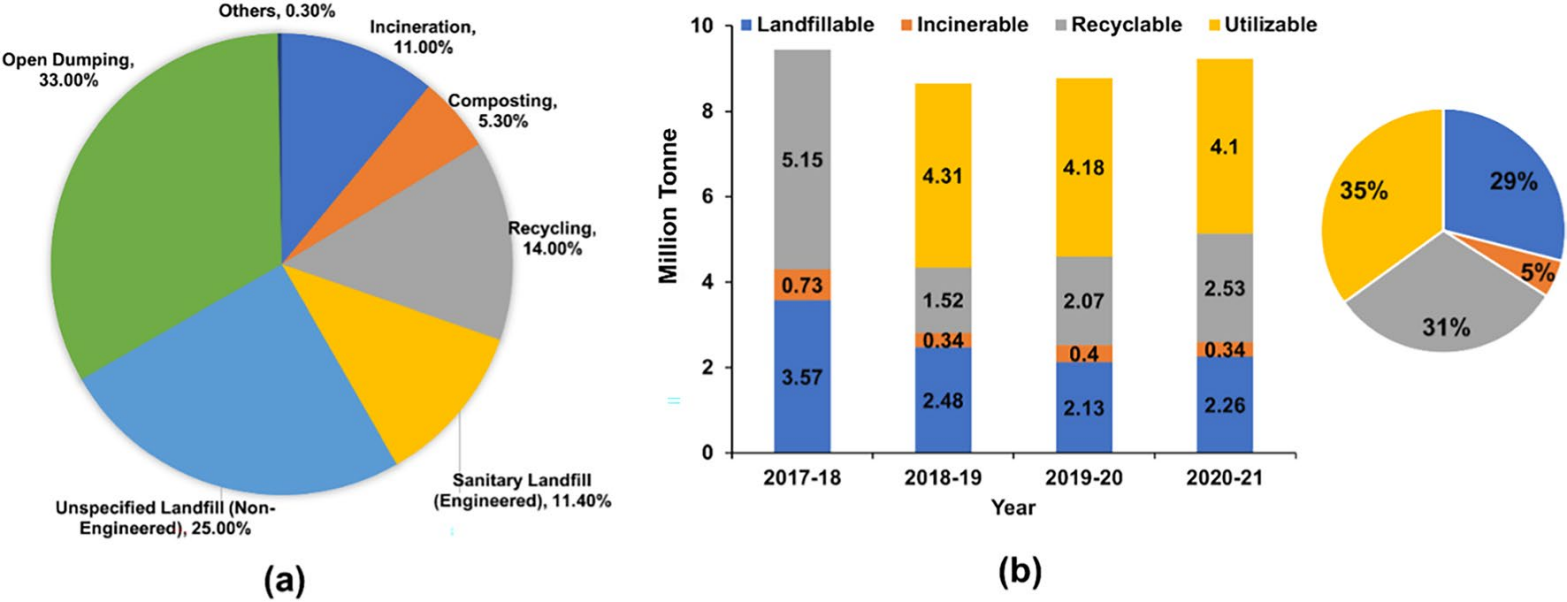
**a** The global status of MSW management (Geyer et al. 2017). **b** Category-wise statistics HW generated between 2017 and 2021 in India (CPCB | inventory 2022)

Figure 9a illustrates the global status of MSW management, and Fig. 9b represents the Indian scenario of the category-wise statistics of HW generated between 2017 and 2021 (CPCB | inventory 2022). The statistics show that between 2017 and 2021, nearly one-third of the HW was landfillable and incinerable, whereas the remaining two-thirds were recyclable and utilisable. Recent literature on common sustainable waste management strategies is presented in Table 3.

**Table 3:** Common sustainable strategies for waste management

Waste management strategy	Waste products	Comments	Reference
Recycling	E-waste	Material flow, economic, and LCA analysis of household appliances (washing machine, refrigerator, cathode ray tube television, fan) was carried out	(Arain et al. 2022)
Recycling	Waste by-products of various sectors	Sustainable recycling of waste by-products such as agricultural waste, construction industry waste, plastics, rubber waste, and marine waste was reviewed	(Junaid et al. 2022)
Recycling	Waste tyre	The waste tyre was used to prepare the carbon anode for lithium-ion (Li-ion) batteries	(Veldevi et al. 2022)
Recycling	Tannery fleshing waste	Preparation of retaining agent for leather processing	(Puhazhselvan et al. 2022)
Recycling	Polyvinyl chloride (PVC) microplastics	Proposed an advanced recycling scheme of PVC microplastics by froth floatation method based on the selective hydrophilisation for the separation of rigid PVC microplastics from flexible PVC	(Zhang et al. 2022a)
Recycling	Waste tyre	Carbon composite derived from the waste tyre was used to prepare the Na-ion batteries	(Li et al. 2016)
Recycling	Hazardous water treatment sludge	Calcined water treatment sludge was used to prepare Portland cement composites for construction application	(He et al. 2021)
Composting	Biomedical waste ash (BMWA)	Vermicomposting was observed to be a sustainable HWM strategy for BMWA	(Sohal et al. 2021)
Composting	Crude oil sludge	Compost bioremediation was reported to be a sustainable technique for hazardous crude oil sludge. Surfactants at different concentrations promoted fungal growth	(Obi et al. 2020)
Composting	Agriculture waste (patchouli bagasse) and cow dung	The vermicomposting end products were enriched with nutrients. The final products were reported to be stable, matured, and free of hazardous materials	(Ahmed and Deka 2022)
Compost-like-outputs	Urban waste	Soil restoration	(Carabassa et al. 2020)
Composting, landfill, incineration, composting, anaerobic digestion, and bioconversion through insects	Food waste	Comparing the treatment strategies for food waste revealed that the incineration and the bioconversion scenarios had the highest environmental benefits. The bioconversion showed better environmental performance compared to composting	(Mondello et al. 2017)
Composting	Coal fly ash	An appropriate combination of buffalo dung, press mud, and fly ash promotes earthworm reproduction and growth	(Karwal and Kaushik 2020)
Home composting	Biowaste	Home composting of biowaste had the potential to achieve sustainable rural development based on a low-carbon society	(Mihai and Ingrao 2018)
Incineration	Medical waste	Enhanced healthcare worker education and standardised medical waste stream sorting are key factors for efficient waste management in healthcare institutions	(Windfeld and Brooks 2015)
Incineration	Halogen and sulphur-containing nano-waste	Emission characterisation was performed for the incinerated waste	(Dutouquet et al. 2022)
Incineration	Municipal solid waste	The LCA analysis revealed that refuse-derived fuel production and incineration could improve the global warming potential from − 33 to 0%, acidification potential from − 90 to 34%, and eutrophication potential from − 1200 to 350% as compared to the co-incineration of MSW with coal	(Havukainen et al. 2017)
Incineration	Municipal solid waste	The review of municipal solid waste incineration	(Makarichi et al. 2018)

### Waste to value-added products

Hazardous waste (HW) consists of biodegradable and non-biodegradable components with high calorific values (Karpan et al. 2021) and huge potential to generate value-added products and be utilised as an alternative to fossil fuels (Sadala et al. 2019). The thermal treatment (pyrolysis, gasification, and combustion) (Dhyani and Bhaskar 2018) and hydrothermal treatment (hydrothermal gasification and liquefaction) (Kumar et al. 2019) are the most favourable approach to reproducing cleaner renewable energy (Okolie et al. 2019) from hazardous waste (Nzihou et al. 2019). The generated value-added products can be either in the form of gaseous (H_2_, CH_4_, and syngas), liquid (bio-oil, ethanol, and chemicals), or solid (biochar) products. The thermal and hydrothermal conversion processes can convert waste into valuable products and simultaneously reduce the burden of their disposal.

#### Pyrolysis

Pyrolysis is a thermal degradation process of organic compounds without air or oxygen. It is one of the promising technologies for converting waste into valuable products (syngas, bio-oil, and biochar) (Kumar and Reddy 2022a). The fast-pyrolysis has gained much attention due to selectively producing high-yield bio-oil at moderate temperature in a very short residence time. Some of the HWs, such as plastics, biomass, tyres, medical waste, household waste, and MSW, are potential feedstocks for pyrolysis to generate energy (Chew et al. 2021). The valuable chemical products (toluene, xylene, aromatic benzene, and ethylbenzene) were produced from the waste polyethylene with the help of catalytic pyrolysis at 700 °C in a semi-batch reactor. They obtained a maximum 78.20 wt% liquid yield (Gaurh and Pramanik 2018). The pyrolysis of waste tyres was performed, and the transformation of nitrogen, sulphur, and chlorine was studied during pyrolysis (Cheng et al. 2021). A pyrolytic thermal degradation study of MSW was performed and able to convert around 84–88 wt% of MSW into bio-oil (26–42%), gaseous product (42–60%), and biochar (12–16%) (Suriapparao et al. 2022).

Pyrolysis has been proved to be an advanced waste of valuable product conversion technology. The solid waste must be dried before pyrolysis. The presence of moisture in the feedstock can result in the generation of char and tar, which lowers the product’s yield and process efficiency. This process has scalability issues due to operational problems such as coking and clogging (Wang et al. 2021). The techno-economic analysis and life cycle assessment of the pyrolysis of urban MSW (unsegregated) showed that the process is energy sustainable if the moisture content is less than 20% (w/w) (Chhabra et al. 2021).

#### Gasification

Gasification is a thermochemical degradation process that converts carbonaceous components into gaseous form, predominately syngas (H_2_ and CO) (Chanthakett et al. 2021). This is one of the most promising techniques to valorise waste material (Hameed et al. 2021). The process is carried out over a temperature range of 800–1200 °C. At an industrial scale, the process is generally initiated auto thermally by reacting the carbonaceous material with a stoichiometric balance amount of oxygen (Shahabuddin et al. 2020). Globally, 114 gasification projects are operational (Munir et al. 2019) (27 projects are on hold and 13 are under construction) and dedicated to electricity generation (106), liquid fuel production (24), gaseous fuel production (8), and chemical production (7) (Molino et al. 2018). In 4 plants, syngas is used for both power generation and fuel production.

The catalytic gasification of HWs (COVID-19 face mask) in the presence of Ni catalyst supported on zeolites (ZSM-5 types) has been reported (Farooq et al. 2022). They reported that an increase in temperature from 600 to 800 °C led to a reduction in carcinogenic compounds and a significant increase in H_2_ production (Farooq et al. 2022). The gasification of industrial hazardous waste (oil sludges) with steam in a tube furnace reactor has been operated in the temperature range of 600–900 °C (Chu et al. 2021). The maximum H_2_ yield was obtained at 800 °C with oil sludge to the steam ratio of 0.3:1. The study based on the catalytic gasification of MSW has reported that after the addition of Ni-CaO-TiO_2_ catalyst (Ni loading ~ 20%), the concentration of H_2_ increased from 35.1 to 57.7%, the yield of dry gas increased from 0.75 to 1.74 Nm^3^ kg^−1^, whereas the tar content reduced from 9.38 to 2.55% (Irfan et al. 2019). The formation of tar and char results in the clogging of the reactor. The thermal gasification of hazardous waste is efficient when the ash and moisture content are very low (ash, 10–15% and moisture content, < 10%). Thermal gasification also has heat transfer limitations (Czajka 2021).

#### Hydrothermal treatment

Supercritical fluid (SCF) based techniques are promising for treating municipal solid waste, sewage, and contaminated water (Kumar and Reddy 2022b). The agricultural and industrial waste (bagasse, mosambi peels, and banana pseudo-stem) were hydrothermally gasified at subcritical and supercritical water conditions in a batch reactor to generate hydrogen in the presence of a heterogeneous catalyst (Ni, Ru, and Fe) (Kumar and Reddy 2019, 2020). Hydrothermally treated pharmaceutical waste and achieved a TOC removal efficiency of greater than 99.9% using the supercritical water oxidation process (Thakur et al. 2019).

Supercritical water gasification (SCWG) (Gaurav et al. 2020) of the cornstalk depolymerisation residue (CGE—99.2%) biomass and the simultaneous recovery of copper (99.9%) from wastewater were investigated in a batch reactor at 22.5 MPa and 650 °C (Chen et al. 2018). The carbon gasification efficiency (CGE) of 99.2% and copper recovery of 99.9% were reported. The performance of SCWG of landfill leachate in a batch reactor is reported as a maximum H_2_ yield (231.3 mmol/L) at 25 MPa, 500 °C, the residence time of 10 min, and oxidation coefficient of 0.2 (Gong et al. 2018). In another study (Chen et al. 2020), the landfill leachate was treated under SCWG conditions (500–650 °C, 22.5–26 MPa) in the presence of a KOH catalyst. The maximum CGE achieved was about 99.2% and the H_2_ yield of 26 mol/kg (Chen et al. 2020). Hydrothermal treatment is a sustainable approach to extracting valuable products from waste and reducing the volume faster. However, it has higher operational and equipment maintenance costs due to high pressure and temperature operating conditions.

### Hazardous waste treatment technologies

The various HW generated from industries in the form of chemicals (e.g. persistent organic pollutants, pharmaceuticals, pesticides, insecticides, personal care products, and halogenated), heavy metals, salts, and oxyanions contaminate land (Wang et al. 2019) and underground water bodies (Fakhru’l-Razi et al. 2009). Household waste (vegetables and fruits waste, plastics, cloths), e-waste, etc., are the main contributors to municipal solid waste (MSW). When exposed to open dumping or unauthorised disposal, such waste pollutes the land, water, and air due to foul and unbearable smells. The treatment of HW using advanced technologies is essential before their disposal. Different physical, chemical, and biological methods are employed for this purpose. But, the selection of these techniques depends on the pollutants’ characteristics. Every method has advantages and limitations in terms of feasibility, efficiency, environmental impact, cost, etc.

Physical processes are mainly based on the mass transfer of toxic pollutants. The low operational cost, simple design, efficient remediation, and requirement of a negligible amount of chemicals are the major advantages of the physical methods. High energy requirements and high operational costs are the disadvantages of physical processes (Thakur et al. 2022). Some commonly used physical methods are air filtration, adsorption, coagulation, membrane-based technologies, etc. (Thakur et al. 2021). The chemical methods are mainly based on the chemical oxidation of toxic pollutants. The chemical oxidation methods such as advanced oxidation processes (AOPs) seem to be most widely used for the remediation of hazardous pollutants from the waste stream (Mudoi et al. 2021). The AOPs are cost-effective, efficient, and safe for the effective remediation of hazardous contaminants from industrial effluents. The chemical oxidation processes are confined to the laboratory scale, and their scale-up is difficult. The biological methods rely on the decomposition or degradation of hazardous pollutants by microorganisms in an aerobic or anaerobic cycle. The biological techniques are cost-effective, produce less sludge and non-hazardous metabolites, and use less water compared to physical/chemical methods. But the biological processes are slow with poor biodegradability and require an optimally conducive environment for microorganisms (Khan et al. 2022). A compilation of different waste treatment technologies is presented in Table 4.

**Table 4:** Different treatment techniques for waste management

Treatments (type)	Waste products	Comments	Reference
Coagulation (P)	Sulphide and arsenic compounds	The compounds were removed by 0.01 M ferric coagulation. The coagulants were effective in the remediation of turbidity, COD, and dissolved organic matters	(Qiu et al. 2019)
Coagulation and electrocoagulation (P)	Mining wastewater containing cyanide	Iron and aluminium salts were used as coagulants. Iron electrochemical coagulation led to the complete removal of cyanide, whereas aluminium electrocoagulation led to 60% removal of cyanide	(Mamelkina et al. 2020)
Electrocoagulation (EC) (P)	Industrial liquorice processing wastewater	EC using Fe rod electrodes led to 94.6% colour removal	(Abbasi et al. 2022)
Adsorption (P)	Pb (II)	Synthesis of magnetic sludge-biochar modified with amino groups adsorbents. The maximum adsorption capacity was 127.0 mg/g	(Huang et al. 2019)
Adsorption (P)	Al(III), Fe(II), and Mn(II) were removed from coal mine-impacted water	Linde type-A (LTA) zeolite adsorbent was used. More than 99% removal at optimal conditions	(Lobo-Recio et al. 2021)
Adsorption (P)	Acid yellow 2GL dye	The maximum dye removal was 97% at optimal conditions	(Kannaujiya et al. 2022)
Sand filtration (P)	Total suspended solids, COD, linear alkylbenzene sulfonate	> 98% removal of the pollutants	(Babaei et al. 2019)
Air filtration (P)	Particulates and toxic gases	> 99.90% removal of particulate matter using a protein-functionalised microfiber/protein nanofiber bi-layered air filter	(Liu et al. 2019)
Supercritical fluid (P)	Pharmaceutical waste	The maximum removal efficiency of 80.1% was achieved with excess oxidant at 400 °C for 60 min in a batch reactor	(Mylapilli and Reddy 2019)
Membrane technology/microfiltration (P)	Oil–water emulsion	A ceramic membrane with a 100-nm pore size was fabricated by spraying alumina particles on a fly ash support. The TOC removal efficiency of > 99% was obtained	(Zou et al. 2019)
Membrane technology/ultrafiltration (P)	Sr^2+^, Pb^2+^, Cd^2+^, Cr^6+^, and humic acid (HA)	UiO-66 incorporated polysulfone membrane was highly effective for the removal of the contaminants	(Wang et al. 2022)
Membrane technology/nanofiltration (P)	Heavy metal salts (ZnCl_2_, PbCl_2_, Ni(NO_3_)_2_, and Cu(NO_3_)_2_)	Polyethylenimine (PEI) cross-linked P84 nanofiltration membranes were developed. 24%P84/PEI membrane was effective for selective removal of various heavy metals	(Zheng et al. 2022)
Photocatalytic oxidation (C)	Benzene and toluene	46% and 57% removal of benzene and toluene	(Jafari et al. 2018)
Fenton-like technology (C)	Methylene blue	Fe_3_O_4_@PDA-MnO_2_ as a catalyst and together with H_2_O_2_ as a Fenton-like reagent led to 97.36% dye removal in 240 min	(Pan et al. 2019)
Catalyst Cu/ZSM-5 Fenton-like oxidation (C)	Phenol	92% of TOC removal in 8 h 7% Cu/ZSM-5 completely degraded phenol in 30 min	(Li et al. 2019a)
Microalgae biodegradation (B)	Phenol	> 97% removal using *Chlorella pyrenoidosa*	(Dayana Priyadharshini and Bakthavatsalam 2019)
Fungal biodegradation (B)	O-desmethylvenlafaxine (ODMVFX) and venlafaxine (VFX) (antidepressant drugs)	100% and 70% removal using white-rot fungus *Trametes versicolor*	(Llorca et al. 2019)
Biodegradation of cyanide degrading bacteria (CNB) (B)	Free cyanide	CNB had great potential to degrade free cyanide	(Razanamahandry et al. 2019)

*P* physical methods, *C* chemical methods, *B* biological methods.

## Future recommendations

Globally, industrialisation and urbanisation are essential components for the socio-economic development of modern society. There is a need to implement policies, and clear and flawless guidelines, for sustainable HWM. Public awareness and proper labelling of hazardous materials are important. Collaboration among the stakeholders and the enterprises is vital to tackle the hazardous waste problems in the utmost effective manner. A sustainable and integrated HWM plan is the future demand for promoting economic growth, creating a more resilient environment, and improving the well-being and socio-economic status of the people.

The future HWM strategy should be aligned with the key components of CE, such as prevention, re-use, recycling, and recovery. Among the various HW, e-waste is one of the highest recycling values. Future research must be focused on the reverse logistics of e-waste. A significant number of precious metals (e.g. gold, copper, and cadmium) present in e-waste is a major concern. There is a need to develop technologies that can reduce the content of precious metals in electronic devices. Further, the efficient and economic recovery of precious metals is very important for the sustainable management of e-waste. Future research should be precisely focused on the techno-economic analysis of the e-waste recycling business on an industrial scale. For other waste also, research should be conducted on energy, efficiency, and economic analysis for the recovery of value-added products.

## Conclusion

The generation of HW is directly linked with socio-economic development and income level. The improper handling, storage, treatment, and disposal of HW adversely impact the environment and public health. A sustainable HWM needs effective policies and regulations and their implementation. There is a need to shift towards circular economic models that are sustainable by design, with equal emphasis on economy, environment, and efficiency. The EU recently introduced a circular economy (CE) hierarchy that consists of 10 R’s prioritise redesigning technology, repair, refrain, repurpose, reduce, remanufacture, re-mine, recover, recycle, and reuse. The HW reduction at source/prevention is the most preferred strategy, followed by waste minimisation (through reuse, recycling, composting), recovery/waste to energy, treatment, and disposal. Recycling is a clean approach but this is relatively undeveloped and often requires high investment costs. An economic analysis should be considered before the implementation of recycling. Also, the need is to design products and adopt technologies that can minimise waste generation by re-use and recycling. Composting is a cost-effective method to manage waste containing organic matter but decomposition of organic waste leads to the emission of greenhouse gases (GHGs). So, there should be a provision to capture GHGs and their utilisation in manufacturing chemicals and other valuable products. Incineration is a commonly used technique for the management of toxic pollutants but with the energy it also emits harmful gases. Landfilling approach is associated with the generation of two types of pollutants, leachate and volatile organic compounds (VOCs). Thus, before landfilling, pre-treatment of waste should be accomplished based on the waste composition and the climatic conditions. A waste-to-energy conversion is a promising approach for the conversion of waste materials into value-added products or the recovery of valuable materials. Thermal treatment (e.g. pyrolysis, gasification) and hydrothermal treatment (hydrothermal gasification and liquefaction) are promising techniques to re-produce cleaner renewable energy from hazardous waste. The formation of tar and char are the major issues with thermal treatment. Hydrothermal treatment has limitations of higher operational and equipment maintenance costs due to high operating conditions of pressure and temperature. Most of the treatment technologies are sustainable and effective for specific types of pollutants. In the recent past, treatment technologies (e.g. physicochemical and biological) are integrated (hybrid) to provide cost-effective and environmentally sustainable HWM. The optimal integration in terms of the economic and technological competitiveness of various strategies can be implemented at a commercial scale for waste management. Based on the types and merits of hazardous and non-hazardous waste, thermochemical and biological treatment technologies can be a better solution for the decomposition/degradation of waste material. Simultaneously, these treatment technologies will be able to address environmental remediation as well as generate value-added products in the form of energy and chemicals.

### Supplementary Information

Below is the link to the electronic supplementary material.

Supplementary file1 (DOCX 26 KB)

## Data Availability

Data sharing not applicable to this article as no datasets were generated or analysed during the current study.
