# Supplementary material for: A critical review on sustainable hazardous waste management strategies: a step towards a circular economy
Source: Environ Sci Pollut Res Int. 2023 Sep 19;30(48):105030–55. doi: 10.1007/s11356-023-29511-8 (PMC10579135; doi:10.1007/s11356-023-29511-8)
Supplement: Supplementary file 1 — Supplementary file1 (DOCX 26 KB) [file 11356_2023_29511_MOESM1_ESM.docx]

**A critical review on sustainable hazardous waste management strategies: a step towards a circular economy**

**Supplementary materials**

**Table S.1: Characteristics Wastes Produced from Various Industrial Activities**

| **Industrial Sector** | **Description** | **Typical Waste** |
| --- | --- | --- |
| Mining and quarrying | Extraction, beneficiation, and processing of minerals | Solid rock, slag, phosphogypsum, muds, tailings |
| Energy | Electricity, gas, steam, and air-conditioning supply | Fly ash, bottom ash, boiler slag, particulates, used oils, sludge |
| Manufacturing | Chemical | Spent catalyst, chemical solvents, reactive waste, acid, alkali, used oils, particulate waste, ash, sludge |
|  | Food | Plastic, packaging, carton |
|  | Textile | Textile waste, pigments, peroxide, organic stabilizer, alkali, chemical solvents, sludge, heavy metals |
|  | Paper | Wood waste, alkali, chemical solvents, sludge |
| Construction | Construction, demolition activity | Concrete, cinder blocks, gypsum, masonry, asphalt, wood shingles, slate, metals, glass, and plaster |
| Waste/water services | Water collection, treatment, and supply | Spent adsorbent, sludge |

**Table S.2: Major policies and legislations/targets related to HWM.**

| **India** | | **China** | | **USA** | | **EU** | |
| --- | --- | --- | --- | --- | --- | --- | --- |
| **Year** | **Legislations/Targets** | **Year** | **Legislations/Targets** | **Year** | **Legislations/Targets** | **Year** | **Legislations/Targets** |
| 1989 | HW rules (management & handling) under the provision of the EPA 1986 | 1996 | - Solid waste law (prevention & control of environmental pollution). - HW identification standards | 1965 | Solid waste disposal act | 1973 | 1^st^ Environmental Action Plan (EAP), based on Stockholm Agreement (UN, 1972). Lasted from 1973 to 1977. |
| 2001 | The Batteries (management and handling) rules | 1998 | National list of HWs (First edition) | 1976 | Resource Conservation and Recovery Act (RCRA), MSW is partially regulated by it. | 1992 | Maastricht Treaty (Council of the European Communities, 1992) focus economic dimension of Europe without any specific direction and goals on environmental protection, based on Rio de Janeiro UN Conference (UN, 1992) |
| 2008 | Repealed and replaced by HWM rules of 1989, covering the provision of the Basel Convention (1992) | 2001 | Dangerous waste pollution prevention and control technology policy | 1980 | - Amendment of RCRA, 1976 by solid waste disposal act - The Comprehensive Environmental Response, Compensation and Liability Act (CERCLA) or - "Superfund" (for HW site remediation) | 1997 | - Treaties of Amsterdam (implemented in 1999) - EU directive (Directive 1999/31/EC), emphasized waste landfill |
| 2010 | Amendment of Batteries (management and handling) rules, 2001 | 2002 | HW pollution control standards (amendment of 1996 rules) | 1984 | Amendment of RCRA, 1976 by Hazardous and Solid Waste Amendment (HSWA) | 2000 | Treaty of Lisbon strategy (covering social pillar on sustainability) |
| 2012 | Ban on the import of all HW as identified under the Basel Convention. | 2003 | Medical waste (MW) management regulations | 1986 | Superfund Amendment Reauthorization Act (SARA) | 2008 | - EU directive (2008/98), emphasizes the basic concepts and definitions related to waste management. - Development of several strategies emphasise prevention, reuse, reduction, recycling, energy recovery |
| 2016 | - Hazardous and other waste (management and transboundary movement) rules - Bio-medical Waste (management and handling) rules - e-wastes (management and handling) rules | 2004 | - National plan for the construction of MW and HW disposal facilities - Measures for the administration of HW business licenses | 1989 | US EPA passes an agenda promoting source reduction, recycling, land-filling, composting, and waste combustion | 2015 | - Paris Agreement (global action plan to avoid climate change) - EU commitments towards achieving UN 17 SDGs (Sustainable Development Goals) |
| 2016 | - New legislation was amended four times and finally adopted and replaced the 2008 legislation - Deals with illegal traffic of waste under the hazardous and other waste (management and transboundary movement) rules | 2008 | - National directory of HWs (2008 edition) - Measures for the administration of approval of exports of HWs | 1990 | Clean Air Act Amendment, also had a direct and indirect influence on HWM. | 2022 (End) | Hazardous house waste separate collection scheme |
| 2017 | National HWM strategy | 2012 | - The 12^th^ 5-y plan for prevention and control of HW pollution - Technical specifications for the collection, storage, and transportation of HWs | 1995 | Amendment of Clean Air Act by US EPA, regulating MSW combustors | 2023 (End) | Biowaste separate collection scheme |
| 2018 | - Amendment of biomedical wastes (management and handling) rules - Amendment of e-waste (management and handling) rules | 2013 | Interpretation of the application of criminal cases concerning environmental pollution | 2008 | EPA’s definition of solid waste (DWS) rule | 2025 (End) | Textile waste separate collection scheme |
| 2019 | - Amendment and adaptation of HWM rules of 2016 (according to the Basel Convention) - Plastic waste included as regulated material | 2014 | Revision of the environmental protection law | 2015 | Amendment of EPA’s DWS, 2008 rules. Revised in recycling-related provisions, determination of HW regulation under RCRA subtitle C. | By 2030 | - To achieve and implement UNDPs and SDGs along with all EU actions and policies - Recycling of packaging waste (up to 70%) - Separated recycling for papers and cardboard (85%), ferrous metals (80%), glass (75%), aluminium (60%), plastic (55%), wood (30%) |
|  |  | 2016 | - National directory of HWs (2016 edition) - Technical policy for prevention and control of solid waste pollution by cement kiln - Circular Economy plan (2016 onwards) |  |  | By 2035 | - MSW recycling (up to 65%) - Reduce landfilling (10%) |
|  |  | 2016-2020 | 13^th^ 5-y plan to deal with national and provincial HW standardisation, management, supervision, and assessment |  |  | By 2050 | - European green deal strategy to achieve climate neutrality |
